# Neutralization of IL-8 Prevents the Induction of Dermatologic Adverse Events Associated with the Inhibition of Epidermal Growth Factor Receptor

**DOI:** 10.1371/journal.pone.0039706

**Published:** 2012-06-25

**Authors:** Nannie Bangsgaard, Mischa Houtkamp, Danita H. Schuurhuis, Paul W. H. I. Parren, Ole Baadsgaard, Hans W. M. Niessen, Lone Skov

**Affiliations:** 1 Department of Dermato-allergology, Copenhagen University Hospital Gentofte, Hellerup, Denmark; 2 Genmab, Utrecht, The Netherlands; 3 Genmab, Copenhagen, Denmark; 4 Department of Pathology and Cardiac Surgery, VU University Medical Centre, Amsterdam, The Netherlands; Istituto Superiore di Sanità, Italy

## Abstract

Epidermal growth factor receptor (EGFR) inhibitors are widely used in the treatment of cancer. EGFR-targeted treatment is known to be associated with a high incidence of dermatological adverse reactions, including papulopustular rash, which can be dose-limiting and may affect compliance to treatment. Currently, the pathways involved in EGFR inhibitor-induced rash are poorly understood and few treatment options for this adverse event are available. Here, we developed a model for induction of papulopustular rash in healthy human volunteers by subcutaneous injection of the anti-EGFR monoclonal antibody zalutumumab. The injection sites and surrounding skin were evaluated by a dermatologist for the presence or absence of papulopustular rash and skin biopsies were taken to confirm the macroscopical findings by immunohistochemistry. Locally injected zalutumumab induced a papulopustular rash, characterized by acute follicular neutrophil-rich hair follicle inflammation, and thus mimicked adverse events induced by systemic administration of EGFR inhibitors. In this model, we tested the hypothesis that neutrophils, attracted by IL-8, play a central role in the observed rash. Indeed, concomitant local repeat dose treatment with HuMab-10F8, a neutralizing human antibody against IL-8, reduced the rash. Inhibition of IL-8 can therefore ameliorate dermatological adverse events induced by treatment with EGFR inhibitors.

## Introduction

Cancer therapy is increasingly shifting towards targeting specific pathogenic pathways. Epidermal growth factor receptor (EGFR; ErbB1) controls proliferation and maturation of epithelial cells in skin. In many solid tumors of epithelial origin, EGFR is up-regulated, making it an attractive target for treatment [1], [2], [3]. Indeed, inhibitors of EGFR, including both small molecules and monoclonal antibodies (mAb), represent a known example of targeted therapy, and are widely used in daily oncologic clinical practice [4]. EGFR inhibitors are less likely than traditional cytotoxic chemotherapeutics to cause myelosuppression, infection, vomiting and nausea. However, several dermatological adverse events accompany the use of EGFR inhibitors. These adverse events affect the patient's well being, may be dose-limiting and influence treatment compliance. A papulopustular (also called acneiform) skin rash is a common toxicity observed with both EGFR-targeting mAb and tyrosine kinase inhibitors (TKI), with a reported incidence of up to 80% in patients treated with EGFR-targeting agents [5], [6], [7].

The rash induced by EGFR inhibitors typically appears within one to three weeks of treatment and is characterized by inflammatory follicular papules and pustules. The rash is most commonly affecting the face; but is also seen at the upper chest and back and infrequently at other body sites [8]. The rash appears to be dose-related [9], [10], and is reversible upon withdrawal of treatment, but may re-appear or worsen once treatment is resumed. Higher response rates and a significant correlation with increased survival have been observed in patients in whoever rash developed [11], [12]. To ensure that patients can continue to receive treatment at the optimal dose, effective treatment strategies are required to actively manage rash and aid compliance. As yet, there are no standardized treatments for these skin side-effects [13], [14], [15]. A greater understanding of the biological mechanisms responsible for the EGFR inhibitor-induced rash would be highly beneficial for the development of rational and more effective treatment management strategies.

The rash may be related to follicular occlusion due to a lack of epithelial differentiation and epithelial inflammation resulting from release of cytokines as direct results from EGFR inhibition. Because the papulopustular rash is characterized by follicular inflammation with an accumulation of neutrophils [16], [17], [18], we hypothesized that the cytokine IL-8 might play a role in this pathology. Previously, we have shown that treatment of patients with palmoplantar pustulosis (PPP), an inflammatory disease characterized by skin infiltration with neutrophil granulocytes, with a neutralizing monoclonal antibody against IL-8, led to a marked improvement in clinical signs concomitant with a reduction in neutrophil infiltration [19].

**Figure 1:**
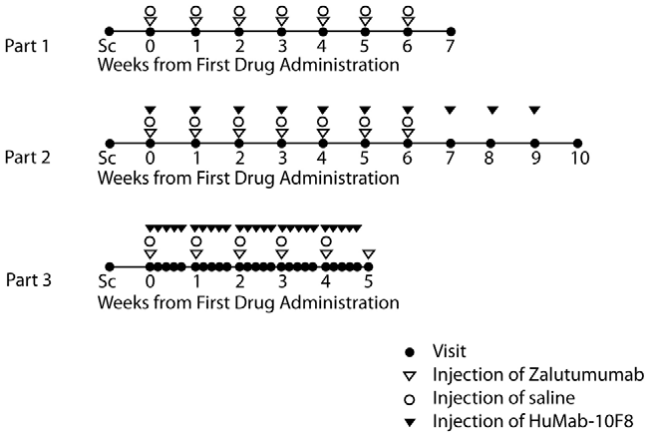
Trial design. Injection schemes for the three different parts of the study are shown. Subjects were injected with different doses of zalutumumab at different sites, as described in Table 1. Sc – screening of patient.

**Table 1:** Dose escalation schedule for zalutumumab injections.

	Visit						
Injection site	Day 0	Day 7	Day 14	Day 21	Day 28	Day 35	Day 42
**1**	1 μg	1 μg	1 μg	1 μg			
**2**		10 μg	10 μg	10 μg	10 μg		
**3**			100 μg	100 μg	100 μg	100 μg	
**4**				1 mg	1 mg	1 mg	1 mg

Every injection of zalutumumab was accompanied by a control injection of an identical volume of isotonic saline at a different site. In the third part the dose escalation started at 100 μg.

**Figure 2:**
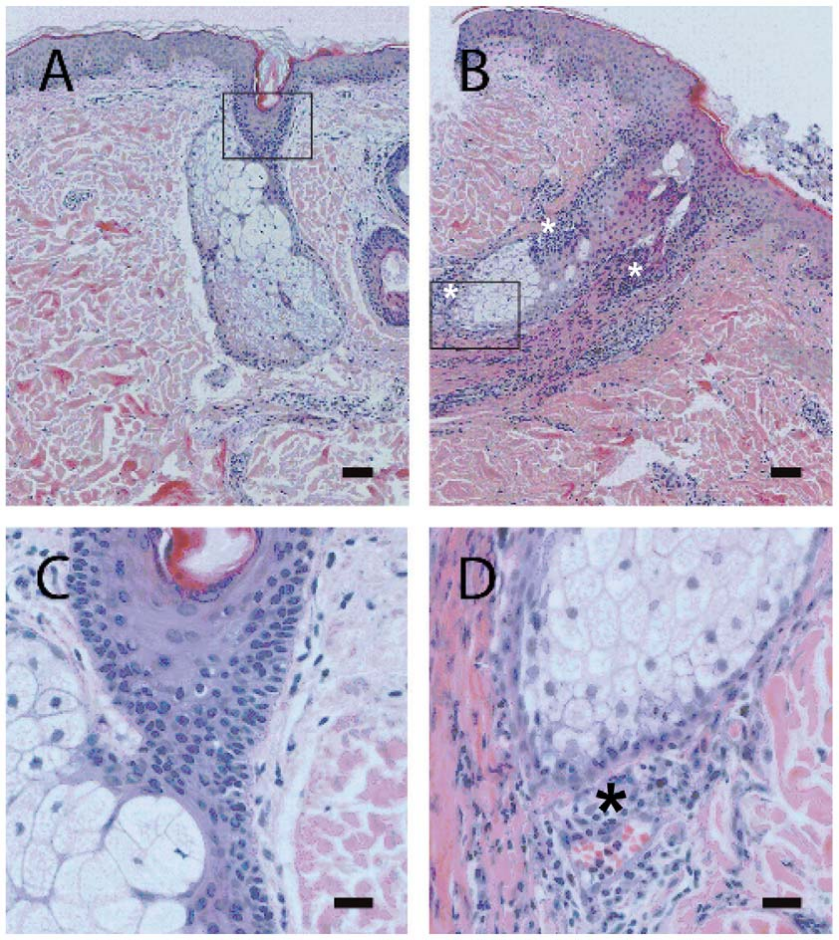
Histology of biopsies from saline- or zalutumumab-injected skin (part I of the study). Representative sections are shown of skin biopsies of one subject for part I of the study (following two injections of 10 μg of zalutumumab). Sections were stained with HE to show nucleated cells and (patho-)histological features. In the saline-injected skin (A, C), inflammatory cells were virtually absent and the hair follicle structure was intact. Note that after zalutumumab injection (B, D), the hair follicle structure was destroyed and extensive influx of inflammatory cells (including neutrophils) in the vicinity of the destroyed hair follicle/glandular structures can be observed (asterisks). Scale bars are 100 µm (A, B) and 20 µm (C, D).

Here we show, in this proof-of-principle study, that inhibition of IL-8 can ameliorate the dermatological adverse events induced with an EGFR-inhibiting mAb. Further studies addressing the potential of IL-8 inhibition for the prevention of serious dermatological adverse events induced both by small molecule as well as biologic EGFR inhibitors are warranted.

## Materials and Methods

An open-label, single-center non-randomized study was performed in healthy volunteers with a single dose escalation set-up. The clinical study was performed at the Department of Dermato-allergology, University Hospital of Copenhagen Gentofte in accordance with the declaration of Helsinki. The study was approved by the local ethics committee (H-KA-20060104) and The Danish Medicines Agency (2006-003253-24). All subjects gave written informed consent prior to enrolment.

**Table 2:** Histological features of hair follicles in skin biopsies and clinical features of patients treated with zalutumumab, zalutumumab + HuMab-10F8 or saline, based on evaluation of HE staining and clinical observations.

		Saline			Zalutumumab			
Patient ID	Study Part	Histology^a)^	#Pustules	Degree of reaction	Dose/µg	Histology	#Pustules	Degree of reaction
2	I	Normal	0	None	1	Acute inflammation	10	Mild
1	I	Normal	0	None	1	Acute inflammation	15	Mild

		Zalutumumab			Zalutumumab + HuMab-10F8			
Patient ID	Study Part	Histology^a)^	#Pustules	Degree of reaction	Dose/µg	Histology	#Pustules	Degree of reaction
3	II	Acute inflammation	2	Mild	100	Acute inflammation	0	Mild
4	II	Acute inflammation	3	Mild	1000	Acute inflammation	5	Mild
2	II	Acute inflammation	1	Mild	100	Normal	1	Mild
5	II	Normal	1	Mild	1	Acute inflammation	2	Mild
1	II	Acute inflammation	1	Mild	1000	Acute inflammation	0	Mild
6	II	Acute inflammation	1	Mild	100	Acute inflammation	2	Mild

		Zalutumumab			Zalutumumab + HuMab-10F8			
Patient ID	Study Part	Histology^a)^	#Pustules	Degree of reaction	Dose/µg	Histology	#Pustules	Degree of reaction
6	III	Normal	1	Mild	1000	Normal	0	None
2	III	Acute inflammation	5	Mild	1000	Normal	3	Mild
5	III	Acute inflammation	3	Mild	100	Normal	0	None
1	III	Acute inflammation	6	Mild	1000	Normal	6	Mild

a) semi-quantitative scoring: normal, acute inflammation (based on influx of neutrophils).

**Figure 3:**
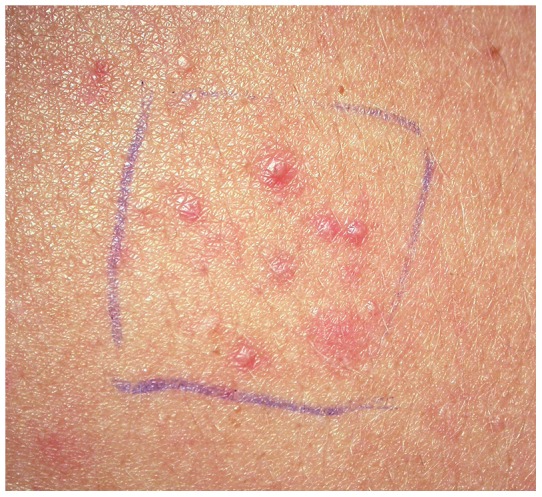
Papulopustular rash was induced by local injection of zalutumumab. The rash observed at the injection site of one subject in part II of the study, following two injections of 100 µg of zalutumumab each, is shown.

**Figure 4:**
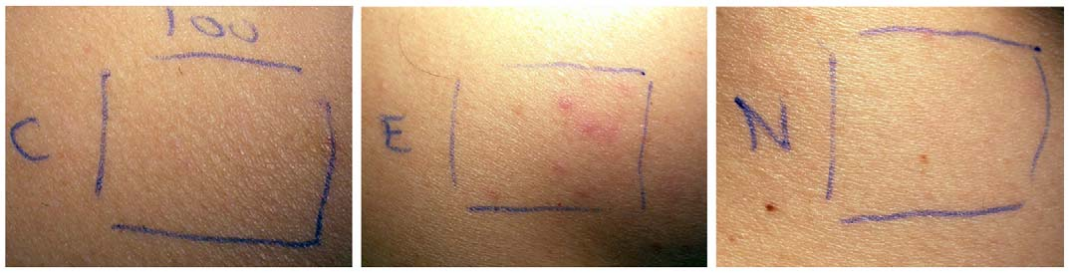
Effect of HuMab-10F8 on zalutumumab-induced pustules. Injection sites of a patient from study part III is shown. Injection of 100 μg zalutumumab alone (E) induced pustules following two injections. Injection of zalutumumab combined with HuMab-10F8, followed by four daily HuMab-10F8 injections (C), suppressed the induction of pustules. The saline control is also shown (N).

**Figure 5:**
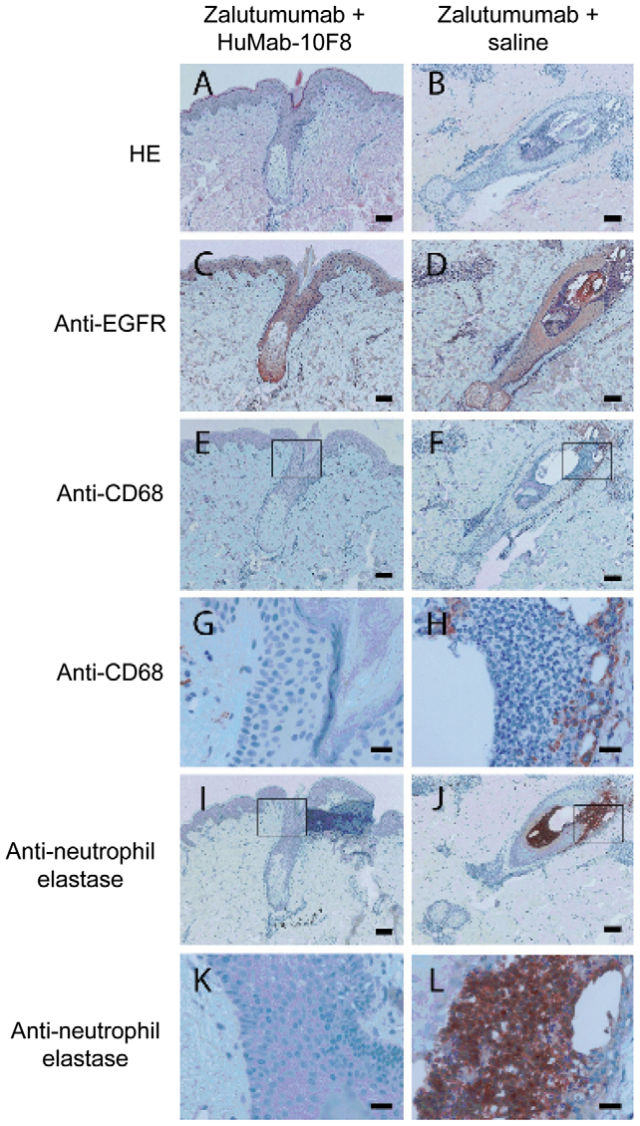
Histology and immunohistochemistry of biopsies from zalutumumab-injected skin treated with HuMab-10F8 or untreated. Data shown are representative sections of skin biopsies of one subject for part III of the study. Biopsies were taken at day 14 after two injections with 100 µg of zalutumumab with [A, C, E, G, I, K] or without HuMab-10F8 [B, D, F, H, J, L]. A, B: HE staining; C, D: staining for EGFR; E–H: staining for macrophages and I–L: staining for neutrophils. Extensive influx of inflammatory cells in the vicinity of the hair follicle with histological signs of folliculitis was observed in zalutumumab-injected skin (right panels), whereas in the zalutumumab-injected skin treated with HuMab-10F8 (left panels) inflammatory cells were virtually absent and sebaceous glands and hair follicles showed normal histological features. Inflammatory infiltrate in the zalutumumab-injected skin was mainly composed of neutrophils (neutrophil elastase positive cells, J, L) and to a lesser extent of macrophages; (CD68 positive cells F, H). Scale bars are 100 µm (A–F, I, J) and 20 µm (G, H, K, L). G, H, K and L are magnifications of the squared areas indicated in E, F, I and J.

A total of nine healthy male volunteers were included in the study. All subjects were Caucasian men and the median age of the group was 24 years (range 22–32 years).

### Injection protocol

The first part of the study was conducted to evaluate whether local subcutaneous (s.c.) injection of zalutumumab could induce a papulopustular rash, similar to that reported in patients treated systemically with EGFR inhibitors. A maximum of four subjects were to be enrolled and attended once weekly for injection of escalating doses of zalutumumab on the upper back. Since there was no experience with s.c. injection of zalutumumab and the local concentration to induce rash was not known, the study was started with a dose-escalation of s.c. zalutumumab (see Table 1 and Figure 1). 1 μg (in 0.2 mL) zalutumumab was injected s.c. on the upper back. The injection site was marked for later identification. One week later, the injection site was inspected macroscopically and scored by a dermatologist for the presence or absence of rash, defined by the appearance of at least one pustule. If there were no signs of pustules, another 1 μg (in 0.2 mL) zalutumumab was injected at the same site. At the same visit, 10 μg (0.2 mL) zalutumumab was injected at another site, marked for later identification. At each visit, a control injection of an identical volume (0.2 mL) of isotonic saline was injected at a different site. The procedure would take place once weekly with a dose-increase of factor 10 each week, over seven weeks or until the subject had a rash considered induced by the zalutumumab treatment. Only one dose level was injected per injection site. Each dose level was applied for a maximum of four weeks or until a rash developed. Part I of the study was performed in a staggered manner (e.g. treatment of subject 2 was only initiated one week after that of subject 1) and this part would be considered completed when at least one volunteer developed a rash.

The second part of the study was conducted to evaluate the effect of the human IL-8 antibody HuMab-10F8 on zalutumumab-induced papulopustular rash. Seven subjects were enrolled. The dose-escalation procedure from the first part was repeated, again starting at 1 μg zalutumumab per dose. Zalutumumab was either injected alone or in combination with 1.25 mg (in 0.25 mL) HuMab-10F8. As a negative control, isotonic saline, in the same volume as the combined zalutumumab/HuMab-10F8 injection (0.45 mL), was injected at a different site at each visit. After the fourth injection of the zalutumumab/HuMab-10F8 combination at one dose level, 1.25 mg HuMab-10F8 was injected alone once weekly for three weeks at the combination injection site (see Figure 1 and Table 1).

In the third and last part of the study, zalutumumab was given in a dose-escalation procedure starting at 100 μg, or 1 mg if the volunteer had received 100 μg earlier in the study. HuMab-10F8 was administered in a repeat dose regimen. Four subjects were enrolled. The procedure was as described for the second part of the study; however, an additional 1.25 mg dose of HuMab-10F8 was administered at four consecutive days after each combined zalutumumab/HuMab-10F8 injection.

### Antibodies

The therapeutic antibodies used in this study were zalutumumab (2F8; HuMax-EGFr); a human IgG1, κ mAb specific for EGFR; generated as described in Bleeker et al., by immunizing HuMAb mice with A431 cells and purified EGFR [20]. The clinical batch was produced by Lonza Biologics (Slough, UK) from CHO DG44 cell line DJT33. Zalutumumab binds to domain III on the EGFR and inhibits tumor growth by a dual mechanism of action: blockade of EGFR signaling and induction of Fc-mediated antibody-dependent cell-mediated cytotoxicity (ADCC) [19]. Systemic administration of zalutumumab was shown to induce rash [21]. HuMab-10F8; a human IgG1, κ mAb specific for IL-8 [19]; the clinical batch MDS001 was produced by Medarex Inc. (New Jersey, US).

### Clinical Assessment

In each study part, injection sites were evaluated once a week and subjects were instructed to contact the investigator as soon as pustules developed. 4-mm punch skin biopsies were taken from a pustule when a rash was considered to be induced by zalutumumab treatment. At the same time, control biopsies were taken from the isotonic saline injection site and, in part two and three of the study, the positive control site (zalutumumab alone).

The investigator assessed the degree of the rash at the marked injection sites using the following scale from 0 to 3: 0: no rash; +1: mild rash; +2: moderate rash; +3: severe rash. The number of pustules at the injection site was counted and photographic documentation obtained.

### Histological and immunohistochemical assessment

Paraffin-embedded skin biopsies were cut at 4 μm thickness, stained with HE and scored as normal or acutely inflamed. Multiple skin sections of each biopsy were examined, to secure that areas with inflammation deeper in the biopsies would not be missed. HE-stained skin biopsies were scored normal when only few diffuse macrophages or lymphocytes were detected in the vicinity of hair follicles (sebaceous glands) and scored acutely inflamed when, in addition to macrophages and lymphocytes, influx of neutrophils was detected (≥50 cells/skin section; semi quantitative score). To confirm the conclusions of the HE staining and identify the inflammatory cells, some skin biopsies were immunostained for EGFR (mouse anti-human EGFR, clone 1F4, cat. # 2239S, specific for the cytoplasmic domain of EGFR; does not compete with zalutumumab for binding to EGFR; Bioke, Leiden, The Netherlands), or with cell type-specific markers identifying neutrophils (mouse anti-human neutrophil elastase, clone NP57, cat. # M0752; Dako, Glostrup, Denmark) and macrophages (mouse anti-human CD68, clone PG-M1; cat. # M0876; Dako). The primary mouse antibodies were detected with a peroxidase-labeled anti-mouse IgG dextran polymer (Envision-PO™ Dako, Glostrup, Denmark; Powervision-PO™, Immunologic, Duiven, The Netherlands) and visualized with AEC (red staining).

## Results

### Induction of papulopustular rash by local injection of zalutumumab

To set up a human model to study EGFR inhibitor-induced rash, healthy subjects were injected s.c. with the human anti-EGFR antibody zalutumumab. Local injections were used to set up a safe model in human volunteers with limited exposure to the therapeutic antibody.

In part I of the study (Figure 1, Table 1), it was investigated whether s.c. injection of zalutumumab could induce rash. Rash occurred simultaneously in volunteers 1 and 2 on day 14 of the study, which was observed as a mild rash at the 1 μg zalutumumab injection site (i.e. following three 1 μg injections in subject 1 and two injections in subject 2) and a milder rash at 10 μg of zalutumumab (i.e. after two and one 10 μg injections in subjects 1 and 2, respectively). The number of pustules counted is shown in Table 2. Histological examination of skin biopsies from zalutumumab-treated areas showed acute neutrophilic inflammation with follicular localization and signs of disruption of the hair follicles (Table 2, Figure 2B, D), whereas in the saline-treated normal skin, inflammatory cells were virtually absent and the hair follicle structure was intact (Figure 2A, C).

### Effect of HuMab-10F8 treatment on zalutumumab-induced papulopustular rash

In part II of the study, it was investigated whether neutralization of IL-8 by a neutralizing antibody, HuMab-10F8, could inhibit the zalutumumab-induced rash. Zalutumumab and HuMab-10F8 were administered concomitantly, once weekly (Figure 1). Seven subjects (including the two subjects from part I) were included in this part of the study. Six of the seven subjects developed pustules following injection of zalutumumab; one at 1 μg, one at 10 μg and four at 100 μg (an example of the rash in shown in Figure 3, the number of pustules counted at the site of biopsy is shown in Table 2). Subject no. 7 was withdrawn due to an adverse reaction (pharyngitis). No clinical or histological differences were observed between the sites with zalutumumab/HuMab-10F8 co-injection compared to sites injected with zalutumumab alone (Table 2). An explanation for these results might be that we did not reach a sufficient local concentration of the IL-8 antibody to achieve a therapeutic effect. Therefore we extended the study with a repeat dose regimen of HuMab-10F8. Four subjects (including the two subjects who completed part I and II of the study and two subjects who completed part II) were included. Zalutumumab was again injected once a week concomitantly with HuMab-10F8 followed by four additional injections of HuMab-10F8 (Figure 1). All four subjects developed pustules following injection of zalutumumab, one at 100 μg and three at 1 mg. Interestingly, an effect of HuMab-10F8 injection on the zalutumumab-induced papulopustular rash was observed in three of four subjects. Clinically, repeat HuMab-10F8 dosing completely suppressed the induction of pustules in two subjects and partially suppressed it in one subject (Table 2). For the zalutumumab positive control alone, induction of pustules was observed in these same subjects (Figure 4). Histological examination supported the clinical findings (Figure 5, Table 2), except in subject no. 6, who had a clinical rash, but normal histology. An extensive influx of inflammatory cells in the vicinity of the hair follicle and signs of folliculitis was observed in the zalutumumab-treated skin (Figure 5 B, D, F, H, J, L). The influx of inflammatory cells consisted predominantly of neutrophils (Figure 5 J, L) and to a lesser extent of macrophages (Figure 5 F, H). In the skin treated with both zalutumumab and repeat doses of HuMab-10F8, the influx of inflammatory cells was virtually absent and sebaceous glands and hair follicles had normal histological features (Fig. 5 A, C, E, G, I, K).

### Adverse events

Overall, five subjects experienced at least one adverse event (10 adverse events were experienced in total). Headache (two subjects), influenza (one subject), heamatochezia (one subject) and neutrophil count decrease (one subject) were judged as not related to zalutumumab treatment; rash distant from the injection site (one subject), fatigue (one subject), pharyngitis (one subject), acne (one subject) and pruritus (one subject) were judged as related to zalutumumab treatment. No serious adverse events were reported.

## Discussion

Prevention and management of EGFR inhibitor-induced dermatological toxicities is critical for patients on such treatments. Histologically, the papulopustular skin rash of patients systemically treated with EGFR inhibitors for cancer often presents with neutrophil-rich suppurative folliculitis [16], [17], [18]. We set up a new model in humans to mimic this adverse event. We here report histological findings, similar to those observed after systemic treatment with EGFR inhibitors, in the skin biopsies from volunteers treated s.c. with zalutumumab. Therefore, this is a model that is very suitable for studying the mechanism behind the development of papulopustular rash and testing potential treatment modalities. Since the model is localized, it is easy to control, and relatively low doses of potential treatments can be studied with minimal risk to the volunteers. We show here that neutralization of IL-8 can ameliorate the symptoms and histological features or EGFR inhibitor-induced papulopustular rash.

Papulopustular rash is reported in up to 80% of patients treated with EGFR inhibitors, both small molecules and mAb [5], [6], [7]. The papulopustular rash is usually mild and with minimal discomfort [5]; however, serious discomfort and altered quality of life have been reported, resulting in dose reductions or treatment withdrawal [22], [23] (out of an 80% overall incidence, grades 1/2 are usually reported at 60%, grades 3/4 at 5–20% [5]). Discontinuation or interruption of cancer therapy may affect clinical outcome. Therefore, effective treatment of these adverse events is required. EGFR inhibitor-induced dermatological toxicities are mainly treated symptomatically, since the mechanisms behind the induction of papulopustular rash by EGFR inhibitors are not well understood. Different agents known to be effective in the treatment of acne such as drying agents, topical antiseptics, topical and systemic antibiotics, topical and systemic retinoids as well as topical and systemic steroids have been used to symptomatically treat the rash, with varying response [5], [6].

EGFR is expressed on epidermal keratinocytes, hair follicle epithelium and sebaceous glands [24], [25]. Activation of EGFR by its ligands plays a crucial role in keratinocyte proliferation, migration, differentiation and survival, keratinization and development of the hair follicle [26], [27], [28]. EGFR inhibition in skin induces apoptosis in normal keratinocytes, which increases five-fold between day 4 and 12, and which correlates with median time to rash onset in patients [29]. Furthermore, inhibition of EGFR disrupts growth and differentiation of the hair follicle [16]. Murine studies indicate that complete or partial abrogation of the EGFR gene causes significantly altered and thinned epidermis and abnormal colonic mucosa, which suggests that the down-stream signaling cascade for EGFR is essential for normal development of epithelial tissues [30], [31]. Some anti-EGFR mAb, such as RG83852, do not inhibit the tyrosine kinase activity of the receptor. In a phase I study of RG83852, no skin rash was observed at doses that produced a high level of saturation of EGFR *in vivo* [32]. This observation suggests that induction of skin rash requires effective EGFR tyrosine kinase inhibition. However, tyrosine kinase inhibition may be necessary, but not sufficient, for the skin rash to occur.

EGFR blockade has been demonstrated to affect chemokine expression in keratinocytes [33]. Increased chemokine expression after EGFR blockade has been shown to result in enhanced skin inflammation [28]. The induced chemokines could play a role in the inflammation with influx of neutrophils, as observed in the skin biopsies. Several studies have aimed to identify histological and immunohistochemical features of skin in patients treated with EGFR inhibitors [17], [18], [34]. Thus, the role of tumor necrosis factor-α (TNF-α) and interleukin-1 (IL-1) in the development of EGFR inhibitor-associated skin rash was shown and a possible therapeutic role for anti-TNF agents was suggested [35]. It has previously been described that the EGFR-specific antibody cetuximab induced papulopustular eruptions and induced production of IL-1 and TNF-α [36]. Cetuximab and zalutumumab employ similar mechanisms of action. Both IL-1 and TNF-α can induce IL-8 secretion by fibroblasts [37] and IL-1 can induce IL-8 secretion by keratinocytes [38]. IL-8 attracts and activates neutrophils [37], [39], [40], [41]. It was therefore hypothesized that EGFR inhibition may (indirectly) induce the production of IL-8, which contributes to the induction of EGFR inhibitor-induced rash. However, it was also described that activation of EGFR induced IL-8 production in keratinocytes *in vitro* [42]. This emphasizes the difficulty of extrapolating findings obtained with one cell type *in vitro* to a whole organ *in vivo*, in this case the skin, which contains many different cell types that interact with each other, and may respond distinctly. Our exploratory clinical trial allowed us to test our hypothesis in humans *in vivo*.

Previously, we showed that local injection of HuMab-10F8, a therapeutic human antibody against IL-8, induced a marked reduction of disease activity in PPP patients [19]. In the present study, we tested the hypothesis that local injection of this therapeutic antibody was capable of reducing the papulopustular rash induced by EGFR inhibition. Repeat dose treatment with HuMab-10F8 was shown to reduce papulopustular rash induced by local zalutumumab injections. The effect was not seen when HuMab-10F8 was applied as a single dose once a week. Because of the local nature of the dermatological events, histological data were only used to illustrate that the zalutumumab-induced rash mimicked the adverse events induced by systemic administration of EGFR inhibitors and to examine the composition of the infiltrate.

In addition to a role in inflammation, IL-8 was also demonstrated to play a role in the survival of tumor cells. Blocking IL-8 has been shown to prevent angiogenesis and reduce tumorigenesis of human non-small cell lung cancer in SCID mice [43]. Furthermore, the combination of antibodies targeting IL-8 with those targeting EGFR resulted in increased anti-tumor effects, as compared with anti-EGFR alone, in a metastatic human breast carcinoma model in SCID mice [44]. Thus, anti-IL-8 and anti-EGFR might provide an interesting combination treatment regimen.

In summary, we developed an innovative human model to study dermatological adverse events induced by EGFR inhibitors. Using this model we showed that neutralization of IL-8 by specific mAb is capable of ameliorating these adverse events. Our study provides a proof-of-principle for a role of IL-8-induced neutrophil migration in the biological mechanisms involved in EGFR inhibitor-induced rash.
